# Atherosclerotic renal artery stenosis is prevalent in cardiorenal patients but not associated with left ventricular function and myocardial fibrosis as assessed by cardiac magnetic resonance imaging

**DOI:** 10.1186/1471-2261-12-76

**Published:** 2012-09-18

**Authors:** Mireille E Emans, Karien van der Putten, Birgitta K Velthuis, Jan JJ de Vries, Maarten J Cramer, Yves GCJ America, Hans L Hillege, Louis Meiss, Pieter AFM Doevendans, Branko Braam, Carlo AJM Gaillard

**Affiliations:** 1 Department of Cardiology, UMC Utrecht, the Netherlands; 2 Department of Nephrology, Leiden University Medical Centre, Leiden, the Netherlands; 3 Department of Radiology, UMC Utrecht, the Netherlands; 4 Department of Radiology, Meander MC Amersfoort, the Netherlands; 5 Department of medicine, division of Nephrology and Immunology and department of Physiology, University of Alberta, Edmonton, Canada; 6 Department of Cardiology, UMC Groningen, University of Groningen, UMC Groningen, the Netherlands; 7 Department of Cardiology, Rijnstate Hospital, Arnhem, the Netherlands; 8 Department of Internal Medicine, Meander MC Amersfoort, the Netherlands; 9 Department of Nephrology, VU University Medical Centre, Amsterdam, the Netherlands

**Keywords:** Cardiorenal failure, Atherosclerotic renal artery stenosis, Magnetic resonance imaging, Late gadolinium enhancement

## Abstract

**Background:**

Atherosclerotic renal artery stenosis (ARAS) is common in cardiovascular diseases and associated with hypertension, renal dysfunction and/or heart failure. There is a paucity of data about the prevalence and the role of ARAS in the pathophysiology of combined chronic heart failure (CHF) and chronic kidney disease (CKD). We investigated the prevalence in patients with combined CHF/CKD and its association with renal function, cardiac dysfunction and the presence and extent of myocardial fibrosis.

**Methods:**

The EPOCARES study (ClinTrialsNCT00356733) investigates the role of erythropoietin in anaemic patients with combined CHF/CKD. Eligible subjects underwent combined cardiac magnetic resonance imaging (cMRI), including late gadolinium enhancement, with magnetic resonance angiography of the renal arteries (MRA).

**Results:**

MR study was performed in 37 patients (median age 74 years, eGFR 37.4 ± 15.6 ml/min, left ventricular ejection fraction (LVEF) 43.3 ± 11.2%), of which 21 (56.8%) had ARAS (defined as stenosis >50%). Of these 21 subjects, 8 (21.6%) had more severe ARAS >70% and 8 (21.6%) had a bilateral ARAS >50% (or previous bilateral PTA). There were no differences in age, NT-proBNP levels and medication profile between patients with ARAS versus those without. Renal function declined with the severity of ARAS (p = 0.03), although this was not significantly different between patients with ARAS versus those without. Diabetes mellitus was more prevalent in patients without ARAS (56.3%) against those with ARAS (23.8%) (p = 0.04). The presence and extent of late gadolinium enhancement, depicting myocardial fibrosis, did not differ (p = 0.80), nor did end diastolic volume (p = 0.60), left ventricular mass index (p = 0.11) or LVEF (p = 0.15). Neither was there a difference in the presence of an ischemic pattern of late enhancement in patients with ARAS versus those without.

**Conclusions:**

ARAS is prevalent in combined CHF/CKD and its severity is associated with a decline in renal function. However, its presence does not correlate with a worse LVEF, a higher left ventricular mass or with the presence and extent of myocardial fibrosis. Further research is required for the role of ARAS in the pathophysiology of combined chronic heart and renal failure.

## Background

The combination of chronic heart failure (CHF) and chronic kidney disease (CKD) is prevalent and associated with a high cardiovascular mortality and morbidity [1-3]. Both CHF and CKD can be caused by atherosclerosis. Renal artery stenosis is often of atherosclerotic aetiology and can manifest itself by hypertension, progressive renal dysfunction, flash pulmonary oedema as well as congestive heart failure [4-6]. Furthermore, it is often diagnosed without evident clinical symptoms. The prevalence of atherosclerotic renal artery stenosis (ARAS) is prevalent in cardiovascular diseases; its prevalence is approximately 15% in patients with proven coronary artery disease and it is up to 40% in patients with end stage renal disease [7]. However, only very few studies have determined the prevalence of ARAS in patients with combined CHF and CKD.

In CHF, in several observational studies, patients with ARAS had prolonged hospital admissions and a higher mortality rate [8-10]. This finding is in agreement with reports that the presence of ARAS is associated with systemic atherosclerosis [11], reduced renal filtration and perfusion and with cardiac abnormalities. However, few data are known about its association with cardiac abnormalities. In an echocardiography study the majority of patients with proven ARAS had cardiac abnormalities, mostly consisting of diastolic dysfunction and left ventricular hypertrophy [12]. To our knowledge, no data are known about its possible association with the presence and extent of myocardial fibrosis, potentially reflecting an increased risk of sudden cardiac death. Although the concurrence of ARAS and cardiac abnormalities may play a role in the cardiorenal syndrome, there are few data to substantiate this and the prevalence of ARAS in combined CKD and CHF is unknown.

We hypothesized that ARAS is prevalent in patients with combined CKD and CHF and that its presence is associated with a worse renal function, more severe cardiac dysfunction and the presence and extent of myocardial fibrosis. Therefore we assessed, in anaemic patients with combined heart and renal failure: 1. The prevalence of ARAS and 2. The association of ARAS with renal and cardiac dysfunction and the presence of myocardial fibrosis. We used cardiac magnetic resonance imaging (cMRI) for this purpose, as the quantitative values for cardiac volumes, left ventricular mass and function are more accurate than with echocardiography. In addition, intravenous gadolinium contrast material can be used both for magnetic resonance angiography (MRA) of the aorta and the renal arteries, as well as for late gadolinium enhanced (LGE) assessment of myocardial fibrosis, thereby providing information about the underlying aetiology of the cardiac dysfunction.

## Methods

### Patient population

This study is a sub study of the EPOCARES study (ErythroPOietin in the CArdioREnal Syndrome, ClinTrials.Gov NCT 00356733). The study design has been published elsewhere [13]. In short, the EPOCARES study is an open-label, prospective, randomized trial, in which patients with CHF, CKD (glomerular filtration rate (GFR) by Cockcroft-Gault equation of 20–70 ml/min) and mild anaemia (haemoglobin 10.3-12.6 g/dL for men and 10.3-11.9 g/dL for women) were included to test the erythropoietic and non-erythropoietic responses to erythropoietin (EPO) treatment. At baseline all patients without cardiac implantable electronic devices underwent combined cMRI and magnetic resonance angiography (MRA) of the renal arteries. During the study period, the concern about the association between certain gadolinium agents and nephrogenic systemic fibrosis surfaced, which led us to the decision to perform cMRI/MRA only in patients with a GFR >30 ml/min. All patients were on maximal tolerated dosages of a β-blocker, angiotensin-converting enzyme (ACE) inhibitor and/or an angiotensin receptor blocker according to CHF guidelines. CHF was defined as NYHA class II or III, based on symptoms and signs [14]. Both patients with heart failure with reduced ejection fraction (HFREF) and patients with heart failure with preserved ejection fraction (HFPEF) were included. HFPEF is defined according to recent guidelines [15]. Hypertension was defined as the presence of a persistent systolic blood pressure >140 mmHg, a diastolic blood pressure >90 mmHg or the use of medication for the treatment of elevated blood pressure in combination with a previous made diagnosis of hypertension. The aetiology of heart failure was divided in ischemic, hypertensive, valvular or other. Ischemic aetiology was defined as having previously had a myocardial infarction, a percutaneous coronary intervention, a surgical coronary artery revascularization, a stenosis of >70% in an epicardial vessel on coronary angiography or the presence of ischemia on nuclear testing. The Medical-Ethical Committee of both the University Medical Centre Utrecht and the Meander Medical Centre (no. 05/220) approved the protocol of the study. Procedures were in accordance with the Helsinki Declaration and all patients gave written informed consent.

### Magnetic Resonance Imaging: acquisition protocol

Cardiovascular Magnetic Resonance Imaging and magnetic resonance angiography (MRA) of the renal arteries were performed on a 1.5 Tesla Philips Intera (Philips Medical Systems, Best, the Netherlands). Both the heart and the renal arteries were assessed in a 45-minute protocol. The patient was placed in the supine position with a five-channel phased array coil for the cardiac analysis and a circularly polarized spine coil in longitudinal direction over the area of the kidneys below the cardiac coil for MRA of the renal arteries.

ECG-triggered breath hold multiphase steady-state free procession (SSFP) images were acquired in the four-chamber, short-axis, and two- chamber view scans of the left ventricle. The short axis plane covered both ventricles from apex to base using 8-mm slices without interslice gap with the following scan parameters: TR/TE 4.0/2.0 ms, flip angle 50, FOV 350–400, matrix 256x256, voxel size 1.6x1.6x8.0 mm.

Gadolinium-based contrast (Dotarem, Geurbet, France) was administered intravenously to first obtain MRA of the renal arteries at the time of injection (first pass) and 15 minutes later delayed enhancement scans of the heart. For MRA of the renal arteries a standard breath hold 3D T1 contrast-enhanced MRA technique was used. Scan parameters: TR/TE 3.7/1.33 ms, flipangle 25, FOV 430x430x75, voxel size 0.8x0.8x1.5 mm.

For the delayed enhancement of the heart, breath hold inversion recovery T1 pulse images were acquired in four-chamber, short axis and left two-chamber view. Scan parameters: TR/TE 4.4/1.3 ms, flipangle 15, FOV 410x410x80, matrix 300x169, voxel size 1.4x1.4x5.0 mm. There were no complications related to the MRI procedures, and all patients tolerated the procedure well.

### Magnetic Resonance Imaging: analysis

The SSFP cine short axis scans were used to acquire measurements of the left ventricle end diastolic volume (EDV), end systolic volume (ESV), ejection fraction (EF) and wall mass (EasyVision release 4 cardiac package, Philips Medical Systems). Endocardial and epicardial contours were traced manually on the stack of contiguous short-axis cine-images at end-diastole. This technique has been validated, with high accuracy and reproducibility [16]. A trained investigator (ME) performed the quantitative image analyses.

Assessment of segmental wall motion and late gadolinium enhancement (LGE) was performed by two independent investigators, blinded for clinical data (BV and YA). The left ventricle was divided in 17 segments according to standardized nomenclature [17] and described as either normal, hypokinetic, akinetic, dyskinetic or aneurysmatic. Enhancing areas of the myocardial wall were identified as fibrotic areas, and separated into probable previous myocardial infarctions (subendocardial to transmural location) or non-ischemic enhancing wall abnormalities (midwall or subepicardial location). To quantify the extent and/or transmurality of the scar tissue, we used the following definitions; a. spatial extent; the number of affected segments; b. transmurality; the number of affected segments with hyperenhancement score of 3 or higher and; c, the total scar score; the summed segmental scores per patient divided by 17 (reflecting the damage for each patient) [18]. Late enhancement was estimated by using a 5-group classification according to the degree of left ventricle wall involvement with 0, absence of hyperenhancement, 1, hyperenhancement of 1% to 25% of left ventricle wall thickness; 2, hyperenhancement of 26% to 50%; 3, hyperenhancement extending from 51% to 75%; 4, hyperenhancement extending 76% to 99% and 5, hyperenhancement extending 100%.

The number of renal arteries as well as patency and presence or absence of stenosis was assessed by two independent investigators, blinded for clinical and cMRI data (JV and LM). Stenosis was graded as: no stenosis, <50% stenosis, 50-70% stenosis, 70-99% stenosis, occlusion 100% or previous stent placement. The presence of a renal artery stenosis was defined as having a stenosis >50% or previous renal revascularization, conform criteria from the Stenting in Renal dysfunction caused by atherosclerotic Renal Artery Stenosis [19].

### Statistical analysis

Data are presented as medians with inter-quartile ranges (IQR) for non-normally distributed variables and means ± standard deviation (SD) for normally distributed continuous variables. Differences between groups were compared with the *χ*^2^ test, Mann–Whitney *U* test or the Kruskal-Wallis one-way ANOVA when appropriate. Differences were considered significant when P < 0.05. For statistical analyses the Statistical Package for Social Sciences (IBM, Chicago, Illinois, USA) version 18 was used.

## Results

### Clinical characteristics

The original study population of the EPOCARES study comprised of 62 patients. Five patients withdrew their informed consent and one patient was excluded due to a suspected malignancy (diagnosed on routine X-ray at baseline). Of the 56 patients that eventually participated in the study, 37 patients underwent a cMRI/MRA. Nineteen patients did not undergo cMRI/MRA due to presence of cardiac implantable electronic devices (n = 15), orthopnoea (n = 2), claustrophobia (n = 1) or a GFR < 30 ml/min (n = 1). These data are obtained after run in treatment with optimal medical therapy for CHF and oral iron supplementation, but before treatment with EPO. Clinical characteristics of these 37 patients are shown in Table 1. The clinical characteristics of the patients that underwent cMRI/MRA did not differ from those patients that did not undergo cMRI/MRA. Overall, patients had markedly reduced eGFR and LVEF. The majority of patients were using a renin-angiotensin blocker and a beta-blocker. A substantial fraction had hypertension and/or diabetes mellitus.

**Table 1:** Clinical characteristics of patients with combined chronic heart failure and chronic kidney disease that underwent MR study

**Variable**	**All patients (n = 37)**	**Atherosclerotic renal artery stenosis**		**p-value**
		**Present (n = 21)**	**Absent (n = 16)**	
Age, years	74 [68–80]	74 [70–79]	74 [60–82]	0.83
Male sex, no. (%)	23 (62.2)	13 (61.9)	10 (62.5)	0.97
Body mass index (kg/m2)	26.5 ± 4.2	26.3 ± 3.5	26.8 ± 5.1	0.84
Creatinine, umol/L	190 ± 81	204 ± 83	172 ± 77	0.19
Cockcroft Gault (ml/min)	37.4 ± 15.6	33.1 ± 12.3	42.9 ± 18.1	0.07
Haemoglobin (g/dL)	11.7 ± 0.82	11.7 ± 0.81	11.7 ± 0.86	0.58
CRP (mg/L)	5 [1.0-10.5]	5 [1.5-9.5]	4.5 [1.0-11.3]	0.95
hsCRP (mg/L)	4.0 [1.3-9.9]	3.8 [1.3-8.3]	5.8 [0.7-10.4]	0.89
NTproBNP (pg/mL)	1400 [621–2499]	1680 [653–2229]	1360 [503–2853]	0.71
Micro albuminuria (mg/24 h)	21.0 [10.3-218.0]	41.5 [14.5-230.0]	12.5 [8.5-93.8]	0.25
SBP, mmHg	145 ± 21.8	150 ± 21.6	138 ± 20.9	0.12
DBP, mmHg	75 ± 11.5	76 ± 13.2	72 ± 8.6	0.23
24-h SBP, mmHg	127 ± 15.3	126 ± 13.1	128 ± 18.3	0.73
24-h DBP, mmHg	66 ± 8.2	66 ± 8.3	66 ± 8.3	1.00
No. of antihypertensive drugs	3.5 [3.0-4.0]	3.3 [3.0-4.0]	3.8 [2.3-5.0]	0.37
RAS inhibitor				
n (%)	36 (97.3)	21 (100.0)	15 (93.8)	0.25
% of recommended dose/day	50 [38–100]	50 [50–100]	75 [25–138]	0.80
β-blocker use, no. (%)	30 (81.1)	16 (76.2)	14 (87.5)	0.38
Diuretic use, no. (%)	29 (78.4)	16 (76.2)	13 (81.3)	0.71
Loop diuretic, no. (%)	24 (64.9)	12 (57.0)	12 (75.0)	0.26
Loop diuretic, dose/day*	40 [0–80]	40 [0–40]	40 [20–110]	0.17
Aldosterone antagonist, no. (%)	5 (13.5)	3 (23.8)	2 (12.5)	0.42
Statin use, no. (%)	28 (75.7)	15 (71.4)	13 (81.3)	0.49
Diabetes, no. (%)	14 (37.8)	5 (23.8)	9 (56.3)	**0.04**
Hypertension, no. (%)	29 (78.4)	18 (85.7)	11 (68.8)	0.21
Smoking history, no. (%)	24 (64.9)	15 (71.4)	9 (56.3)	0.38
Pack years	16.4 [0–31]	16.4 [0–33]	11.5 [0–30]	0.34
Cerebrovascular disease, no. (%)	7 (18.9)	6 (28.6)	1 (6.3)	0.11
Peripheral arterial disease, no. (%)	14 (37.8)	11 (52.4)	3 (18.8)	0.05
Kidney length (cm) (n = 34)	10.9 ± 4.7	10.3 ± 2.2	11.5 ± 6.5	0.85
Aetiology of heart failure:				0.58
Ischemic, no. (%)	22 (59.5)	13 (61.9)	9 (56.3)	
Hypertensive, no. (%)	5 (13.5)	3 (14.3)	2 (12.5)	
Valvular, no. (%)	4 (10.8)	3 (14.3)	1 (6.3)	
Other, no. (%)	6 (16.2)	2 (9.5)	4 (25.0)	
NYHA class				0.21
II, no. (%)	27 (73.0)	17 (80.9)	10 (62.5)	
III/IV, no. (%)	10 (27.0)	4 (19.0)	6 (37.5)	

Mean ± standard deviation or median [interquartile range] is shown.

Abbreviations; CRP, C-reactive protein; hsCRP, high sensitive CRP; NTproBNP, N-terminal pro-brain natriuretic peptide; SBP, systolic blood pressure; DBP, diastolic blood pressure; RAS inhibitor, renin-angiotensin-system inhibitor; NYHA, New York Heart Association class for heart failure.

· Loop diuretic dose/day of furosemide, bumetanide was converted to 1 mg bumetanide = 40 mg furosemide.

### Renal artery stenosis

**Figure 1:**
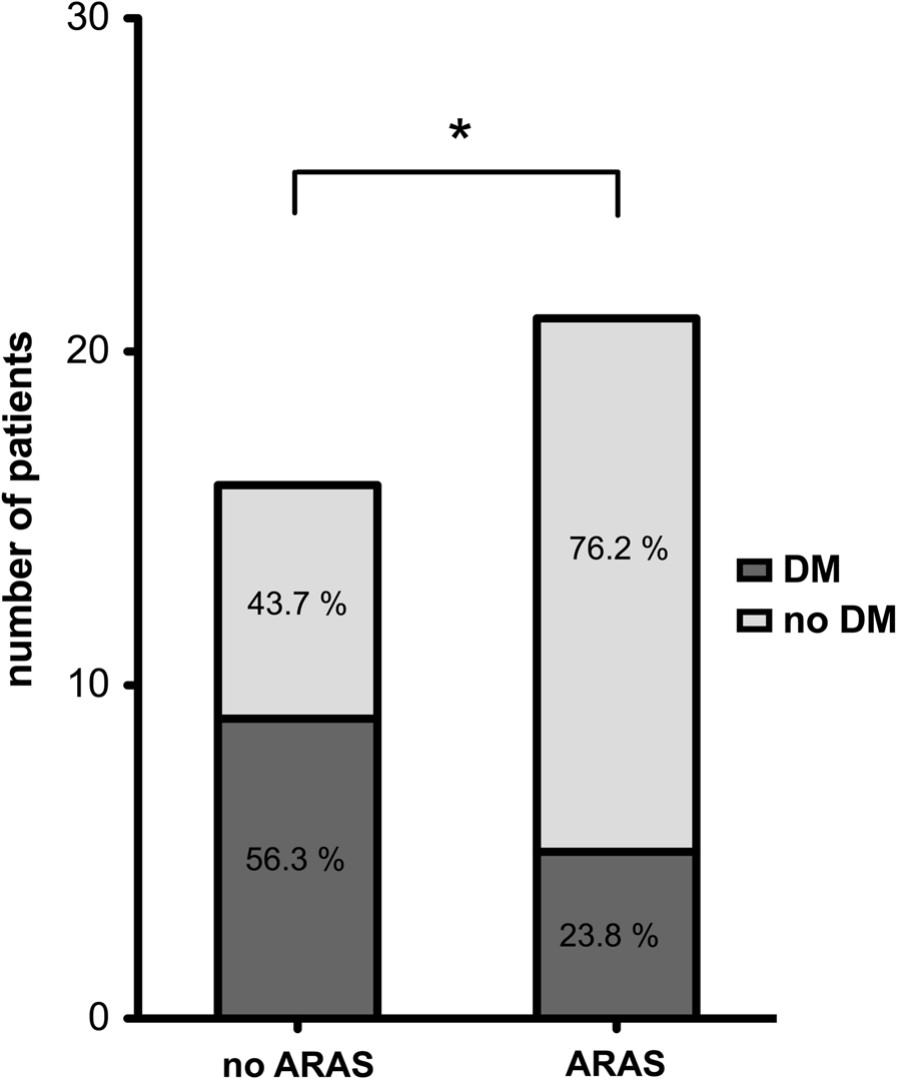
Number of patients with and without diabetes mellitus (DM), divided by the presence of atherosclerotic renal artery stenosis (ARAS) in patients with combined chronic heart failure and chronic kidney disease.*p = 0.04.

**Figure 2:**
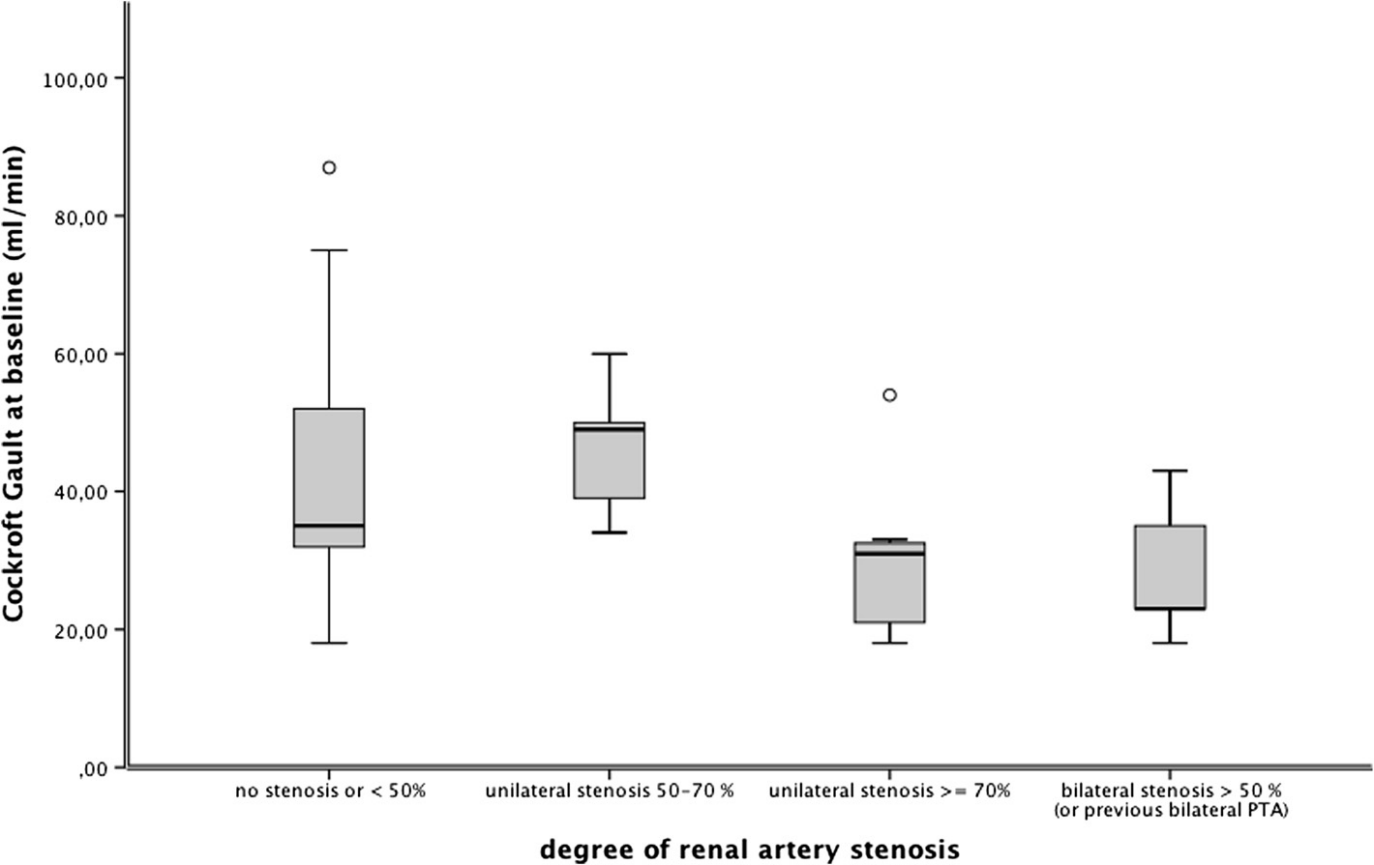
**Comparison of Cockcroft Gault equation (ml/min) in patients with combined chronic heart failure and chronic kidney disease, divided by increasing severity of atherosclerotic renal artery stenosis.** (Shown: median and interquartile range).

Of the 37 patients that underwent cMRI/MRA, 21 patients (56.8%) had a renal artery stenosis defined as >50% stenosis. A more severe stenosis, defined as >70%, was present in 8 (21.6%) patients. A bilateral ARAS (>50%) was present in 7 (18.9%) patients. All stenosis were of atherosclerotic origin. One patient (2.7%) was previously treated bilaterally by angioplasty with stent placement. Baseline demographic, clinical and laboratory characteristics of the patients, divided up by the presence or absence of ARAS, defined as >50% stenosis, are provided in Table 1. There were no differences in age, sex, smoking, the amount of pack years, and the aetiology and severity of heart failure (NYHA class and NTproBNP levels) between patients with ARAS versus those without ARAS. Although there seems to be a tendency for a higher systolic blood pressure based on the office measurements, there is no statistically significant difference in the averaged 24-hour ambulatory blood pressure measurements. The number of antihypertensive drugs in patients with and without ARAS did not differ. Diabetes mellitus was significantly more prevalent in patients without ARAS (Figure 1). We did not find a significant difference in renal function when patients with and without ARAS were compared. Nonetheless, the renal function did significantly decline as the severity of ARAS increased, (ANOVA; p = 0.03); patients with a unilateral ARAS of >70% or with bilateral ARAS had a lower GFR (Table 2 and Figure 2). However, there were no other differences in clinical profile between patients with a moderate ARAS (>50%, less than 70% stenosis) versus those with a more severe ARAS (>70% stenosis) (data not shown). The doses/day for RAS inhibition and loop diuretics were not different between patients with and without ARAS. There were also no apparent differences in medication profile between patients with more or less severe ARAS.

**Table 2:** Clinical variables of patients with combined chronic heart failure and chronic kidney disease, according to severity of atherosclerotic renal artery stenosis by magnetic resonance angiography

	**No stenosis (n = 16)**	**Unilateral stenosis 50-70% (n = 5)**	**Unilateral stenosis >70% (n = 8)**	**Bilateral stenosis >50% or previous bilateral PTA (n = 8)**	**p-value**
Cockcroft Gault (ml/min)	42.9 ± 18.1	46.4 ± 10.2	30.1 ± 11.3	27.9 ± 8.9	**0.01**
RAS inhibitor:					
No. (%)	15 (94)	5 (100)	8 (100)	8 (100)	0.72
% of recommended dosage/day	75 [25–138]	50 [38–125]	58 [31–100]	50 [50–100]	0.99
diuretic use, no. (%)	13 (81)	3 (60)	7 (88)	6 (75)	0.68
Loop diuretic, dosage/day^*^	40 [20–110]	40 [0–120]	40 [10–40]	10 [10–70]	0.43

Mean ± standard deviation or median [interquartile range] is shown.

Abbreviations: RAS inhibitor, renin-angiotensin-system inhibitor.

* loop diuretic dose/day of furosemide, bumetanide was converted to 1 mg bumetanide = 40 mg furosemide.

### Cardiac magnetic resonance imaging

Data regarding cMRI are shown in Table 3. Although the average LVEF by cMRI was 43.3% in the study population, 8 patients (21.6%) had an LVEF >50%, underlining the fact that many patients with combined CHF and CKD have HFPEF. There were no differences between patients with versus those without ARAS regarding LVEF, left ventricular volume or the left ventricular mass index. LGE was present in 27 of the 37 patients (73%). In 24 of these 27 patients the LGE was subendocardial or transmural, determined as ischemic LGE. Non-ischemic LGE (midwall or subepicardial) was present in 6 patients; 4 patients had hypertensive heart failure, 1 patient had severe valvular disease and 1 patient had previously had a myocarditis. In 2 of the 4 patients with hypertensive heart failure, there was both midwall and subendocardial LGE present, as was the case in the patient with myocarditis. Based on baseline clinical data, only 22 of the 24 patients with ischemic LGE were known with CHF with ischemic aetiology. There was no difference in the presence and the extent of LGE in patients with ARAS when compared to those without ARAS (Table 3). Nor was there a difference between the two groups regarding the presence and the extent of ischemic LGE versus non-ischemic LGE.

**Table 3:** Cardiac magnetic resonance imaging findings in patients with combined chronic heart failure and chronic kidney disease

**Cardiac MRI parameter**	**All patients (n = 37)**	**Atherosclerotic renal artery stenosis**		**p-value**
		**Present (n = 21)**	**Absent (n = 16)**	
LVEF (%)	43.3 ± 11.2	46.1 ± 9.1	40.2 ± 12.7	0.15
LVESV (ml)	101 [81.8 -127.9]	96 [81.7 – 114.4]	115 [81.4 – 177.0]	0.19
LVEDV (ml)	186 [153.8 - 206.2]	173 [156.4 - 202.3]	197 [138.5 - 279.0]	0.60
LV mass index (g/m2)	49 [43.0 - 59.7]	46 [43.2 - 53.3]	60 [41.7 - 69.0]	0.11
Cardiac output (l/min)	5.4 ± 1.85	5.3 ± 1.16	5.5 ± 2.44	0.72
Cardiac index (l/min/m2)	2.7 ± 0.75	2.7 ± 0.62	2.6 ± 0.90	0.65
**Late gadolinium enhancement**				
Spatial extent	3.5 [1.0 - 6.0]	4.0 [0.5 - 6.0]	3.0 [2.0 - 6.5]	0.65
Transmurality	2.0 [0–4.0]	2.0 [0.0 - 4.0]	2.0 [0.0 - 4.5]	0.89
Total scar score	0.56 [0.12 - 0.99]	0.71 [0.09 – 1.03]	0.35 [0.18 - 0.88]	0.80

Mean ± standard deviation or median [interquartile range] is shown.

Abbreviations; LVEF, left ventricular ejection fraction; LVESV, left ventricular end systolic volume; LVEDV, left ventricular diastolic volume; LV mass, left ventricular mass.

## Discussion

Our study shows a high prevalence (56.8%) of atherosclerotic renal artery stenosis (ARAS) in patients with combined heart and renal failure, defined as having a stenosis >50%. When taken a stricter cut-point (> 70% stenosis) the prevalence of ARAS was still as high as 21.8% in this population. The presence of ARAS was not associated with the extent in abnormalities in left ventricular function or myocardial fibrosis based on cardiac MRI findings with late gadolinium enhancement (LGE), when compared to patients with combined CHF/CKD without ARAS. Neither did we observe a difference in the presence of an ischemic pattern of LGE. Furthermore; we found a weak association between eGFR and the severity of ARAS and observed a negative association between diabetes mellitus and ARAS. This could indicate that our small cohort may consist of two different groups of cardiorenal patients: non-diabetic patients in which ARAS is highly prevalent and diabetic patients with a much lower ARAS prevalence.

ARAS is very common in patients with manifestations of non-renal atherosclerosis, particularly in patients with peripheral arterial and aortic disease. A recent literature review found a pooled prevalence of 25.3% in patients with peripheral arterial disease and 33.1% in patients with aortic aneurysm [7]. Only few, small, studies determined the prevalence of ARAS in patients with CHF. McDowall et al. reported a prevalence of 34% of ARAS in patients with CHF and deSilva et al. found a prevalence of up to 54% [9,20]. In both studies, ARAS was defined as a stenosis of >50% by magnetic resonance angiography (MRA). About the prevalence of ARAS in combined CHF/CKD, even fewer studies are published. DeSilva et al. reported a prevalence of 68% in 97 patients with CHF that had renal dysfunction. The study by deSilva et al. included only patients with HFREF. However, HFPEF is known to have a similar poor prognosis as HFREF and to be more prevalent in older patients and in patients with diabetes and/or hypertension [21,22]. Our study included an ambulant stable outpatient clinic patient population with combined chronic heart failure and chronic kidney disease and mild anaemia. We included patients both with HFREF and HFPEF, treated with renin angiotensin (RAS) inhibitors and β- blockers according to present guidelines. In this cohort we demonstrate a high prevalence of ARAS (56.8%). A more severe degree of ARAS, defined as a unilateral stenosis of >70% and/or bilateral stenosis >50% was found in 43.2% of the patients. These results are similar to those of deSilva et al., confirming this high prevalence of ARAS in combined CHF/CKD in patients with both HFREF and HFPEF.

According to the present American Heart Association guidelines for the management of patients with peripheral arterial disease, the indication for percutaneous renal revascularization with stent placement (PTA) of ARAS is limited to “flash pulmonary oedema, recurrent episodes of unexplained congestive heart failure or unstable angina” [23]. There is debate however whether PTA could benefit CHF patients with ARAS. In patients with stable CKD and/or hypertension it has been reported several times that PTA does *not* affect renal function [5,19,24]. On the other hand, a small retrospective study showed that in patients referred for renal revascularization close to one-third had CHF (mainly HFPEF) and that revascularization was associated with better control of heart failure [25]. The results of the sub analysis of the Angioplasty for Renal Artery Lesions (ASTRAL) study of a predefined group of patients with CKD and reduced ejection fraction are not yet available.

Nonetheless, diagnosing ARAS can be important for more reasons than to find patients suitable for PTA. One could interpret the presence of ARAS as a marker of “atherosclerotic burden” associated with a high risk of cardiovascular events, which would warrant more aggressive medical therapy [8]. Indeed, in a follow up study of elderly people with ARAS, the annual incidence of coronary events, heart failure and death were as high as 30%, 19% and 17% respectively [26]. A recent retrospective study in elderly patients with ARAS presented a very high morbidity and mortality (49% suffered a primary event and 37% died during median follow-up of 3.3 years), which was negatively associated with the use of statins.

In addition to determining the prevalence of ARAS in patients with both CHF and CKD, we determined whether there is an association between left ventricular structure and function and the presence of ARAS. Although one could hypothesize that ARAS would be associated more often, and to a greater extent, with cardiac abnormalities, such as left ventricular hypertrophy, we found no differences in left ventricular mass index, left ventricular volumes and LVEF in patients with and without ARAS. We also hypothesized that the existence of ARAS represents an “atherosclerotic burden”, representing one of the mechanisms of combined CHF/CKD. However, we found no difference in the presence of ischemic aetiology of CHF. Moreover, we could not demonstrate a difference in the presence, the location and the extent of fibrosis, as depicted by LGE. The study by Wright et al. showed more diastolic dysfunction and left ventricular hypertrophy in patients with ARAS when compared with a matched control group with similar renal dysfunction [12]. In contrast, our study only included patients with known CHF and CKD. In those patients with combined CHF and CKD, the presence of ARAS was not associated with a more severely impaired cardiac function, more severe left ventricular hypertrophy or the presence of fibrosis.

Coincidentally we found a negative association between diabetes and ARAS; the patients with combined CHF/CKD without ARAS were markedly more likely to have diabetes mellitus than those patients with ARAS (Figure 1). Some studies identified diabetes mellitus as a predictor for ARAS [27], whereas others showed that diabetes mellitus was not associated with ARAS [28,29]. The negative association between diabetes and ARAS may point to a mechanistic difference in the pathophysiology of combined CHF/CKD in patients with and without diabetes. However, alternatively, the difference may result from survival bias.

A point of debate is the definition of ARAS, since no uniform definition exists. Previously, most studies defined ARAS as >50% stenosis [19,24,30-32]. Indeed, the American Heart Association Guidelines [23] are based on studies using this definition. However, more recent guidelines from the European Society of Cardiology define ARAS as having a stenosis >60% [33], based on the fact that MRA (and CT angiography) tend to overestimate the degree of stenosis. More recent PTA studies often combine this definition with additional haemodynamic measurements, e.g. a systolic pressure gradient [34], measurement of the fractional flow reserve [35] or use a more strict cut-point of >70%. In this study we used the definition of ARAS as defined >50%, in accordance with the STAR study [19]. However, if we also apply the more stringent definition of >70% stenosis we still find a prevalence of 21.8% of ARAS. Except for a significant decline in renal function in patients with a more severe ARAS versus a moderate ARAS, we found no other differences in clinical profile between a moderate or severe ARAS.

Some limitations of this study need to be acknowledged. The small study population, due to the complexity of the study design, consists of a selected group of stable ambulant patients, almost all using RAS inhibitors which, in addition to the exclusion of uncontrolled hypertension and patients with flash pulmonary oedema, may have led to underestimation of the prevalence of ARAS in CRS. However, despite this small study population, we believe that these data are valuable and robust, due to a paucity of data in the present literature about this subject and the reliable assessment with cardiac MRI. Furthermore, the data are baseline data from a randomized intervention study, which precludes spontaneous follow-up.

## Conclusion

In conclusion, ARAS is prevalent in patients with combined CHF and CKD and its presence does not correlate with worse left ventricular function, left ventricular volumes, mass, nor myocardial fibrosis as assessed by MRI. However, the severity of ARAS is weakly associated with renal dysfunction and ARAS is remarkably negatively associated with diabetes mellitus in this cohort. Further research is needed to investigate its role in the pathophysiology of combined chronic heart failure and chronic kidney disease and subsequent therapeutic consequences.

## Abbreviations

CKD: Chronic kidney disease; CHF: Chronic heart failure; ARAS: Atherosclerotic renal artery stenosis; cMRI/MRA: Combined cardiac magnetic resonance imaging with magnetic resonance angiography of renal arteries; NT-proBNP: N-terminal pro brain natriuretic peptide; LGE: Late gadolinium enhancement; LVEF: Left ventricular ejection fraction.

## Competing interests

The authors declare that they have no (financial or non-financial) competing interests.

## Authors’ contributions

CG, BB and KvP initiated this study. ME helped in the study design, wrote the manuscript, together with CG and BB, participated in the recruitment of patients and collection of data, together with KvP, did the statistical analyses with HH and analysed the imaging data. MJ, BK and YA analysed the cardiac data. JV and LM interpreted the MRA of the renal arteries. PD contributed to the study design and the discussion. All authors read and approved the final version of the manuscript.

## Funding

This investigator-initiated study was supported by the Netherlands Heart Foundation (grant number 2005B192) and by an unrestricted grant from Roche Pharmaceuticals, the Netherlands. A Heart and Stroke Foundation of Canada New Investigator award supports Branko Braam. This is an investigator-initiated study and no changes to the study protocol were made upon request of Roche, neither did Roche have insight in the data.

## Pre-publication history

The pre-publication history for this paper can be accessed here:

http://www.biomedcentral.com/1471-2261/12/76/prepub
